# DNA Methylation Patterns According to Fatty Liver Index and Longitudinal Changes from the Korean Genome and Epidemiology Study (KoGES)

**DOI:** 10.3390/cimb44030075

**Published:** 2022-02-27

**Authors:** Young Kyung Ko, Hayeon Kim, Yoonseok Lee, Young-Sun Lee, Jeong-An Gim

**Affiliations:** 1 Division of Pulmonary, Allergy and Critical Care Medicine, Department of Internal Medicine, Korea University Guro Hospital, Seoul 08308, Korea; youngsoka@naver.com; 2 Department of Pathology, Korea University College of Medicine, Seoul 08308, Korea; kimhayeon223@korea.ac.kr; 3 Department of Internal Medicine, Korea University College of Medicine, Seoul 08308, Korea; seok7288@korea.ac.kr; 4 Medical Science Research Center, College of Medicine, Korea University Guro Hospital, Seoul 08308, Korea

**Keywords:** non-alcoholic fatty liver disease (NAFLD), fatty liver index (FLI), differentially methylated region (DMR), longitudinal changes, Korean Genome and Epidemiology Study (KoGES)

## Abstract

The role of differentially methylated regions (DMRs) in nonalcoholic fatty liver disease (NAFLD) is unclear. This study aimed to identify the role of DMR in NAFLD development and progression using the Korean Genome and Epidemiology Study (KoGES) cohort. We used laboratory evaluations and Illumina Methylation 450 k DNA methylation microarray data from KoGES. The correlation between fatty liver index (FLI) and genomic CpG sites was analyzed in 322 subjects. Longitudinal changes over 8 years were confirmed in 33 subjects. To identify CpG sites and genes related to FLI, we obtained enrichment terms for 6765 genes. DMRs were identified for both high (*n* = 128) and low (*n* = 194) groups on the basis of FLI 30 in 142 men and 180 women. To confirm longitudinal changes in 33 subjects, the ratio of follow-up and baseline investigation values was obtained. Correlations and group comparisons were performed for the 8 year change values. *PITPNM3*, *RXFP3*, and *THRB* were hypermethylated in the increased FLI groups, whereas *SLC9A2* and *FOXI3* were hypermethylated in the decreased FLI groups. DMRs describing NAFLD were determined, and functions related to inflammation were identified. Factors related to longitudinal changes are suggested, and blood circulation-related functions appear to be important in the management of NAFLD.

## 1. Introduction

The global prevalence of nonalcoholic fatty liver disease (NAFLD) has increased to 25% of the general population and is the main cause of chronic liver disease [1,2,3]. Although most patients show a benign clinical course [4], 20–33% of patients progress to nonalcoholic steatohepatitis (NASH) with fibrosis, which can develop to liver cirrhosis and hepatocellular carcinoma [5,6]. In addition, NAFLD is associated with elevated risk for cancer development and cardiovascular diseases [7,8,9]. Several factors are associated with the development and progression of NAFLD, including age, sex, genetics, epigenetics, and other metabolic factors [10,11,12]. The relationship between NAFLD development and progression and genetic and epigenetic factors has not been fully elucidated.

Therefore, screening of NAFLD patients in the general population is important to identify patients with a high risk of disease progression. NAFLD can be diagnosed when patients have hepatic steatosis confirmed by liver biopsy and imaging studies such as ultrasonography, computed tomography (CT), and magnetic resonance imaging (MRI) without other secondary causes of hepatic fat accumulation [13]. However, liver biopsy has several limitations, including high cost, complication risk, and inter-/intra-observer variation; therefore, it is not recommended for diagnosing NAFLD [14]. Imaging studies also have limitations for screening in the general population and repetitive examinations due to their high cost. Several serologic biomarkers have been developed, including the fatty liver index (FLI), hepatic steatosis index (HIS), NAFLD liver fat score, and NAFLD ridge score [15]. Among them, FLI showed moderate accuracy in diagnosing NAFLD (AUROC 0.84) and a correlation with prognosis in patients with NAFLD [16,17]. As the FLI is easily calculated using body mass index, waist circumference, triglycerides, and gamma-glutamyltransferase, it can be easily applied in clinical practice and large cohort studies.

To date, several epigenome-wide association studies (EWASs) have been conducted on metabolic traits, including liver diseases [18,19,20]. EWAS is difficult to perform in individual laboratories because DNA methylation analysis must be performed at the genomic level for many samples. Therefore, it is necessary to perform EWASs on follow-up data from large-scale cohorts at the national level. In Korea, a survey during 2001–2016 was obtained from the Ansan and Ansung (AS-AS) cohort of the Korean Genome and Epidemiology Study (KoGES) released by the Korea Center for Disease Control and Prevention [21]. DNA methylation information was obtained for 446 patients in the KoGES baseline survey and for 50 patients in the follow-up survey. Therefore, a systematic analysis of 496 and 50 pairs of baseline and follow-up data, respectively, was performed. To date, EWAS related to smoking [22,23], obesity [24,25], kidney transplantation [26], and diabetes [27,28] have been studied, but studies related to liver disease have not been performed.

DNA methylation reflects the state of health or chronic diseases. Somatic variants reflect cancer-specific changes and gene expression changes reflect various diseases. However, somatic variants can only be analyzed for carcinoma, and it is difficult to use RNA to identify changes in gene expression. Therefore, it is important to extract factors related to health conditions from DNA obtained from the blood and to extract CpG sites that describe health conditions. Hepatic steatosis is an important factor for liver disease progression and is related to the progression of other metabolic diseases, resulting in cardiovascular disease and malignancies [29,30,31]. Evidence of a correlation between DNA methylation and the progression of liver diseases, including NAFLD, has been reported. Therefore, analysis of DNA methylation patterns related to hepatic steatosis may be important in the development and progression of NAFLD [32]. The correlation between DNA methylation and FLI in this study is expected to play an important role in the discovery of biomarkers related to the management of patients with NAFLD. In this study, we attempted to identify FLI-related CpG sites on the basis of the Methylation 450 k BeadChip from normal cohort subjects and their longitudinal subjects.

## 2. Materials and Methods

### 2.1. Subjects and Data Source

The epidemiological and epigenomic datasets of this study were collected from a survey during 2001–2002 and were obtained from the AS-AS cohort of the KoGES released by the Korea Center for Disease Control and Prevention [21]. Participants were 40–69 years of age and belonged to the Ansan and Ansung communities located in Gyeonggi province, South Korea. All the participants provided written informed consent. For DNA methylation data, the matrix provided by KoGES was used, and the detailed bioinformatics process followed has been described in a previous study [33]. This study was approved by the Institutional Review Board (IRB) of Korea University (approval number: KUIRB-2020-0191-01) and was performed in accordance with the Declaration of Helsinki.

### 2.2. Study Design

**Figure 1:**
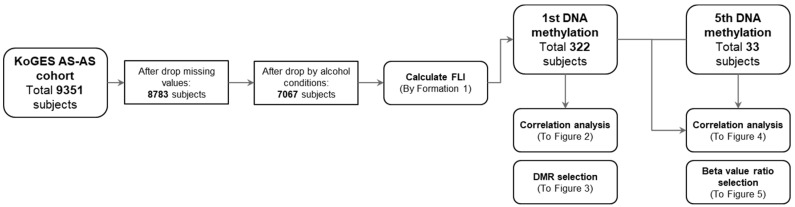
Flowchart of this study. Methylation analysis was performed in blood samples of 446 subjects from the Korean Genome and Epidemiology Study (KoGES) Ansung-Ansan (AS-AS) cohort. Of the 446 samples, 50 samples were reanalyzed at follow-up after 8 years. A total of 322 subjects with the laboratory parameters necessary to calculate the fatty liver index (FLI) were obtained. Among them, samples were available for 33 subjects and were followed up. The correlation between the FLI value and the beta value was obtained and presented as correlation plots for each CpG. On the basis of the FLI value of 30, we selected differentially methylated regions (DMRs) between high and low groups.

In this study, we investigated the epigenetic patterns of NAFLD. We divided the participants into two groups on the basis of one-time (*n* = 322) and longitudinal (*n* = 33) analyses. Flowcharts describe the conditions under which the subjects were excluded from the analysis and classified (Figure 1). The Pearson correlation between the CpG beta value and FLI was measured for the 322 patients included in the primary analysis. The comparison pattern between the beta value and FLI was analyzed over time. As a case–control study, we selected high and low intergroup differentially methylated regions (DMRs) for 322 subjects on the basis of the FLI 30. For the 33 patients included in the longitudinal study, CpG sites were extracted for the group with increased and decreased beta value ratios.

The longitudinal change in FLI was calculated by dividing the baseline by the follow-up. To categorize longitudinal change in FLI, it was divided into an up group when it was more than 1.1 and a down group when it was less than 0.9. Out of a total of 33 subjects, except for 10 subjects, 13 subjects were classified as up and 10 subjects as down. The characteristics of each group are presented in Table 1.

### 2.3. DNA Methylation and Bioinformatics Analysis

In this study, genomic DNA extracted from the peripheral blood of subjects was used to evaluate DNA methylation levels. Genomic DNA (500 ng) from each sample was modified by treatment with sodium bisulfite provided in the EZ DNA methylation kit (Zymo Research, Irvine, CA, USA), according to the manufacturer’s instructions. Genome-wide DNA methylation was profiled using the Illumina Infinium Human Methylation 450 k BeadChip (Illumina, San Diego, CA, USA), which contains over 485,000 CpG probes covering 99% of the RefSeq genes. Each CpG probe has a beta value ranging from 0 to 1; higher methylation of CpG gives rise to a value closer to 1. We analyzed 364,050 CpG probes in 423 subjects, excluding the missing values. Furthermore, the gene symbol and genomic location corresponding to each CpG probe of the Illumina 450 k methylation chip were obtained using the “getAnnotation” function of the IlluminaHumanMethylationEPICanno.ilm10b2.hg19 library provided by Bioconductor (https://www.bioconductor.org, accessed on 25 February 2022).

### 2.4. Bioinformatics Analysis

DMRs were identified using a *t*-test between the high and low FLI in each of the four groups. The filtered DMRs were visualized as volcano plots and heatmaps. Enrichment analyses were performed using DMRs and visualized as KEGG and GO dot plots, gene concept networks, and heatmaps. Enrichment analysis was performed using the “clusterProfiler” and “DOSE” packages. We used the “pheatmap” package to provide heatmaps. For the correlation analysis, we used Python v3.6.9, based on the Google Colaboratory platform and the “corr” function of the “pingouin” package.

## 3. Results

### 3.1. Study Processes

The process followed in our study for the selection, classification, and variant processing of the study subjects is presented as a flowchart in Figure 1. We recruited 9351 participants, and 13 variables were used to process the FLI. When missing values were detected in at least one of the 13 variables, the subjects were dropped. Subjects who consumed excessive alcohol (men > 210 g/week and women > 140 g/week) were excluded from the study. Finally, 7067 subjects were followed up.

DNA methylation analysis was performed on 322 subjects, which is the intersection of 446 subjects whose methylation patterns were analyzed and 7067 subjects who met the filtering criteria. From 446 subjects in the base survey, the methylation of 50 subjects in the fifth survey (fourth follow-up) was re-analyzed. To confirm longitudinal changes, we retrieved 33 subjects, which is the intersection of 50 subjects whose methylation patterns were re-analyzed and 7067 subjects for the filtering criteria. Nineteen demographic and laboratory evaluations, including age, sex, TG, GTP, AST, and ALT, associated with NAFLDs were analyzed. Male participants were classified into the FLI-high group (Table 1).

### 3.2. Correlation Analysis between CpG Site Methylation and FLI

**Figure 2:**
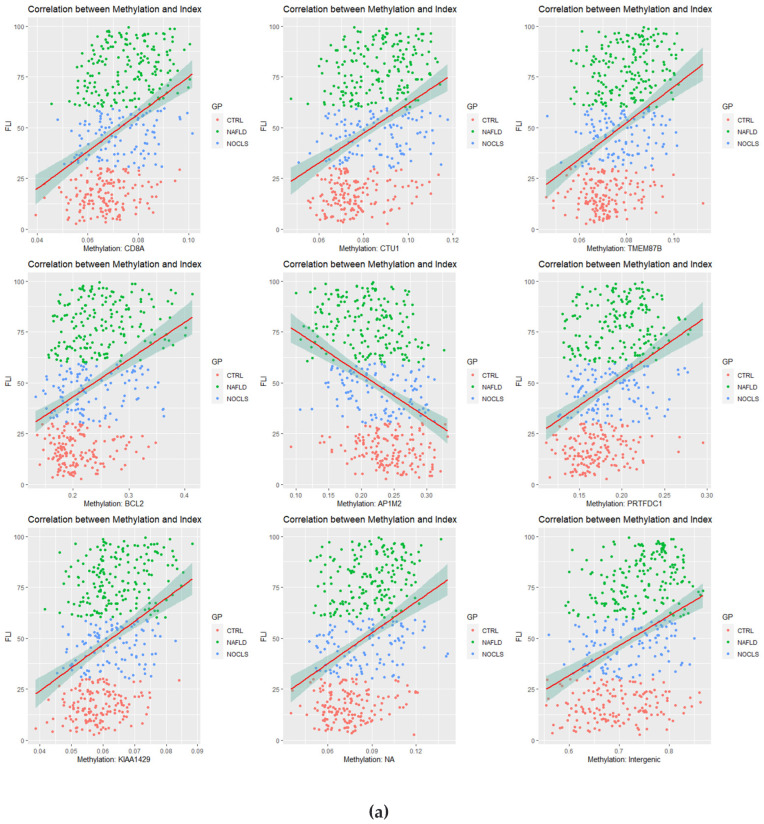

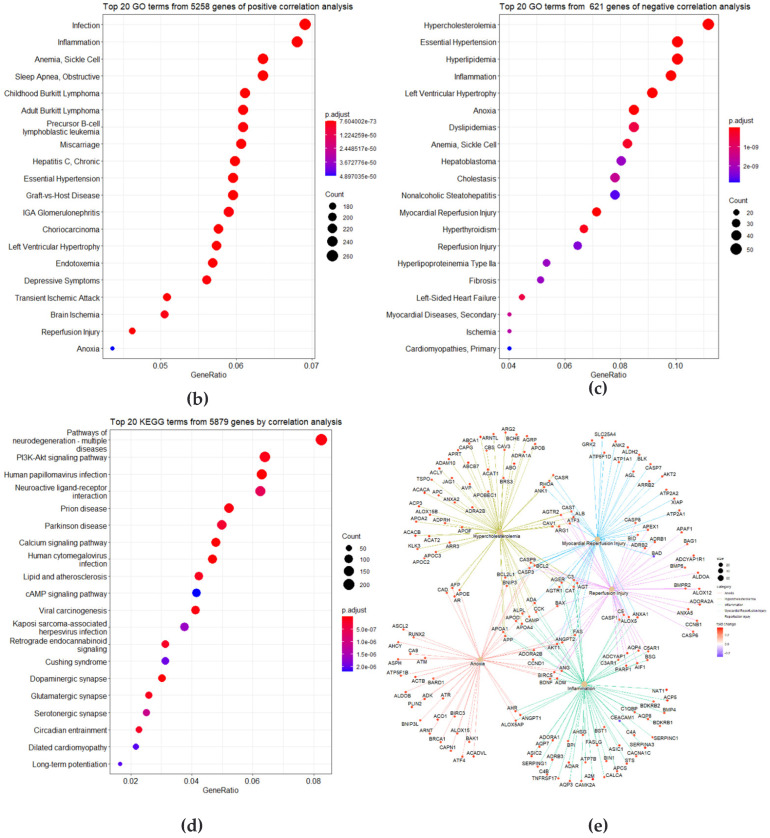
Correlation analysis. (**a**) Correlation plots are listed in the top high nine squares of the correlation coefficients. The plots show β-values of each CpG site (*x*-axis) versus the fatty liver indices (FLIs) (*y*-axis) with regression lines. (**b**–**d**) Enriched gene ontology (GO) terms and Kyoto Gene and Genome Encyclopedia (KEGG) pathways of correlation coefficients between CpG site beta-values and FLIs. Color of the plot represents the *p*-value, and the size of the count of genes. (**e**) Gene-concept network for correlation coefficients was provided. In each network analysis, the top five terms are listed, and related genes are connected. Color scale of the related genes stands for the correlation coefficients. Red and green points indicate correlation and anti-correlation between CpG sites of genes and FLI, respectively.

Pearson’s correlation coefficient was calculated using the beta value of each probe and the FLI. From 403,129 correlation results, 15,628 probes with power >0.99 were selected. Nine genes with the top nine r-squared values were selected and are presented (Figure 2a). Nine CpG probes were retrieved (*CD8A*, *CTU1*, *TMEM87B*, *BCL2*, *AP1M2*, *PRTFDC1*, *KIAA1429*, *cg13188812*, and *cg08257212*). The last two probes were non-annotated (NA) probes (*cg13188812*) located in the intergenic region (*cg08257212*).

Among the duplicate symbols, probes with a low r-squared value and those located intergenically were excluded. Subsequently 5879 gene symbols were identified. GO terms were extracted for 5258 genes with positive correlations and 621 genes with negative correlations, and the top 20 terms are presented as dot plots (Figure 2b,c). The top 20 KEGG terms are shown (Figure 2d). A network analysis was performed on 892 genes with an absolute value of correlation coefficient >0.28, and five terms were connected (Figure 2e).

### 3.3. Identification of DMRs

**Figure 3:**
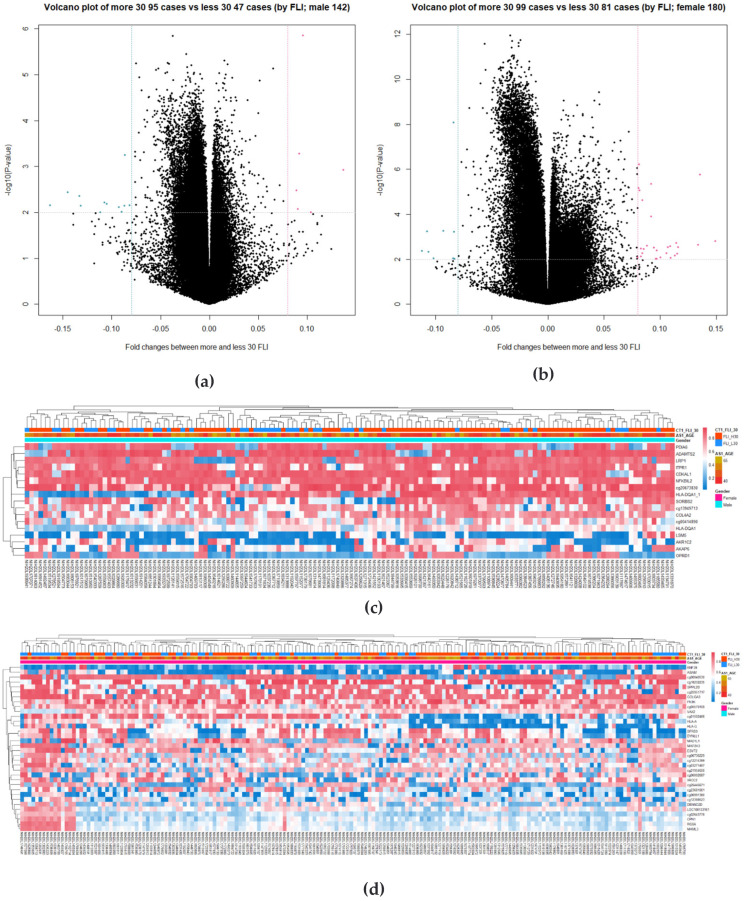

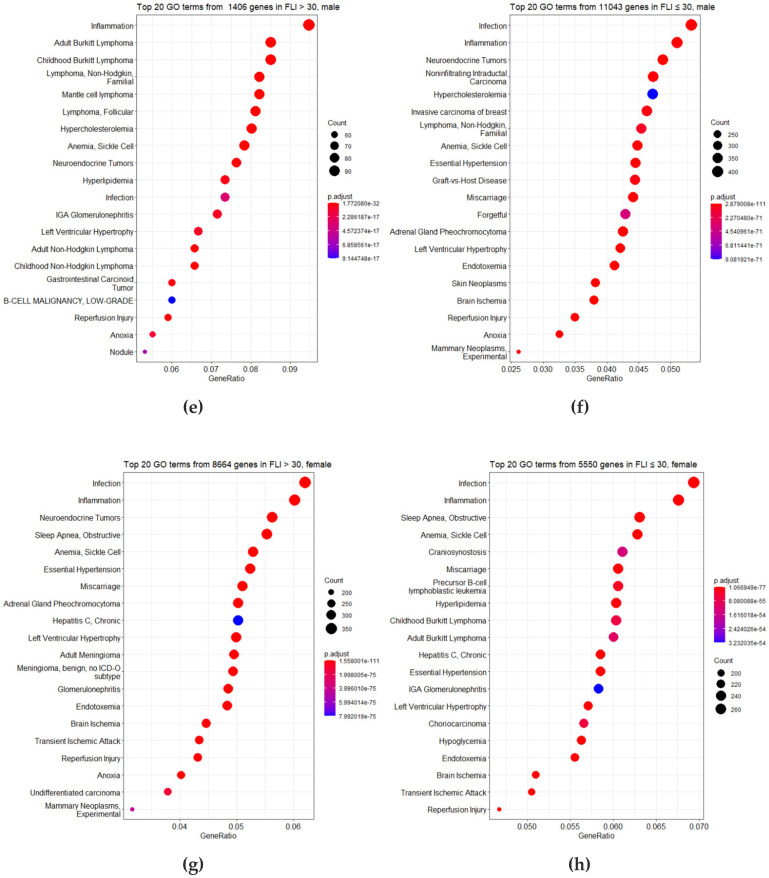

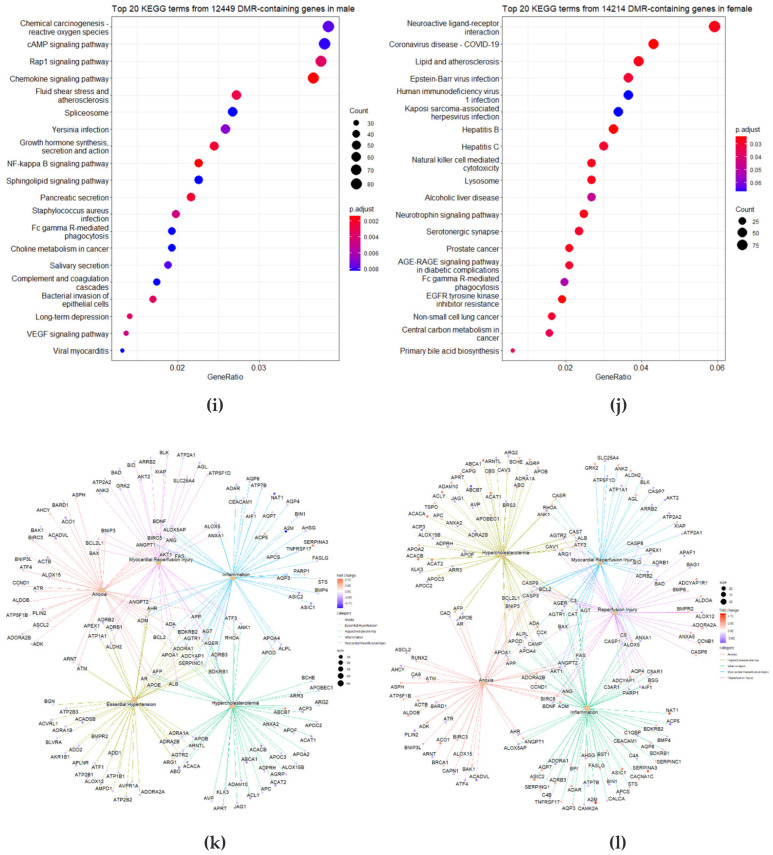
Visualization of differentially methylated regions (DMRs) for each group. *p*-values and fold changes (FCs) were retrieved from the *t*-test for the high and low fatty liver index (FLI) groups. (**a**,**b**) In the volcano plots, total probes are plotted by FC and *p*-value. The condition is |FC| > 0.08, *p*-value < 0.01. (**c**,**d**) Heatmaps are presented for 17 probes in males and 34 probes in females. (**e**–**j**) Enriched gene ontology (GO) terms and Kyoto Gene and Genome Encyclopedia (KEGG) pathways for the high and low FLI groups in each sex. Color of the plot represents the *p*-value, and the size of the count of genes. (**k**,**l**) Gene-concept network for correlation coefficients was provided. In each network analysis, the top five terms are listed, and related genes are connected. Color scale of the related genes stands for the FCs. Red and green points indicate high and low between the high and low FLI groups in each sex.

Because of differences in DNA methylation patterns between female and male subjects, the groups were divided according to sex to avoid bias. We examined the demographic variables to classify the subjects on the basis of the FLI 30 and two sexes for methylation analysis, as shown in Table 1. Volcano plots depict DNA methylation matrices of the two sexes. Points that met the conditions are marked in pink (highly methylated in FLI > 30) and blue (highly methylated in FLI ≤ 30). In each plot, the *x*-axis represents the fold change (FC), and the *y*-axis represents the *p*-value on a −log10 scale. The area that meets the threshold of FC and *p*-value is represented by two vertical lines and one horizontal line. On the basis of these criteria, differences in methylation levels were confirmed between the high-and low-FLI groups in both sexes (Figure 3a,b). Two pairs of DMRs are presented as heatmaps (Figure 3c,d). In each sex analysis, |FC| subject to >0.08 and *p*-value < 0.01, 17 probes for males and 34 probes for females were selected. Heatmaps were generated for the two sexes and showed variation in DNA methylation between the two FLI groups. Column annotation bars indicate three parameters in the samples: FLI group, age, and sex (male and female).

On the basis of the FCs, GO and KEGG terms were retrieved (GO; Figure 3e–h, KEGG: Figure 3i,j). A network analysis was then performed (Figure 3k,l). Five terms were identified for each sex, two of which were common (inflammation and anoxia).

### 3.4. Correlation Analysis of Longitudinal Data

**Figure 4:**
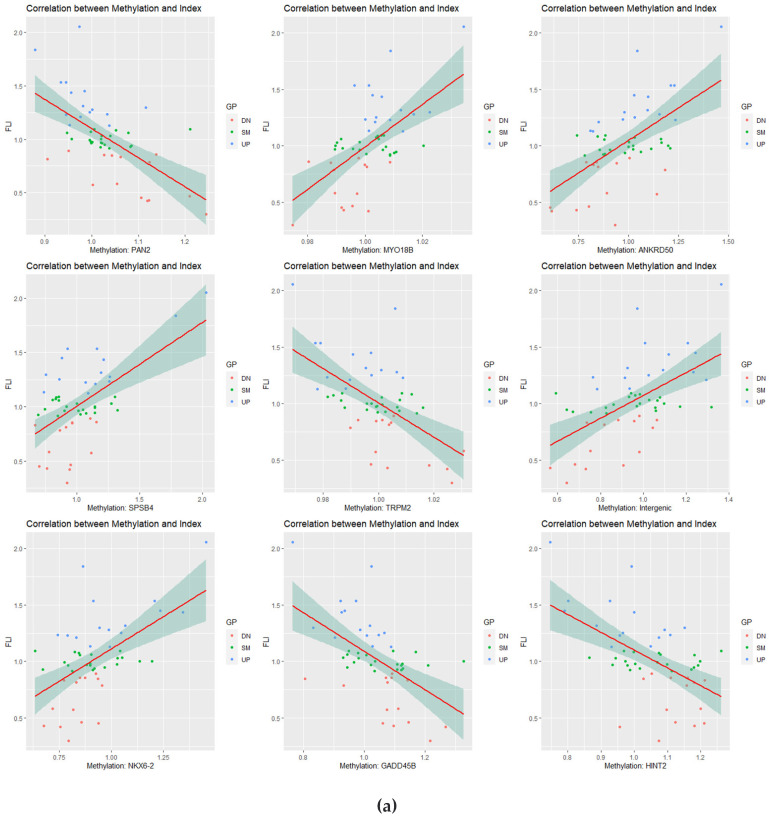

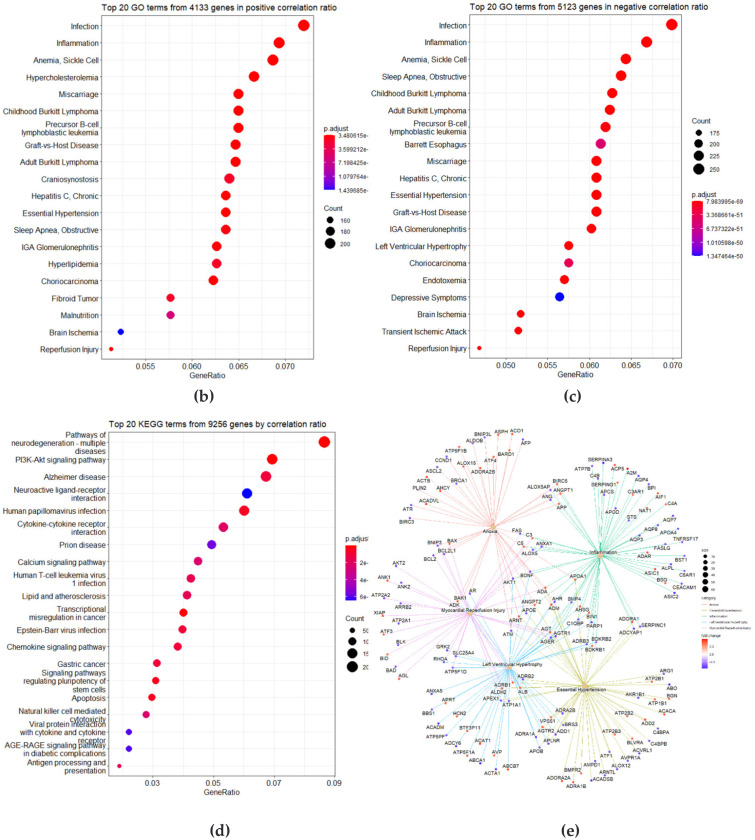
Correlation analysis. (**a**) Correlation plots are listed in the top high nine squares of the correlation coefficients. The plots show β-values ratios of each CpG site (*x*-axis) versus the fatty liver indices (FLIs) ratios (*y*-axis) with regression lines. (**b**–**d**) Enriched gene ontology (GO) terms and Kyoto Gene and Genome Encyclopedia (KEGG) pathways of correlation coefficients between CpG site beta-values and FLIs over time. Color of the plot represents the *p*-value, and the size of the count of genes. (**e**) Gene concept network for correlation coefficients was provided. In each network analysis, the top five terms are listed, and related genes are connected. The color scale of the related genes stands for the correlation coefficients. Red and green points indicate correlation and anti-correlation between CpG sites of genes and FLI over time, respectively.

To confirm the longitudinal change in DNA methylation, we divided the follow-up by the beta value at baseline. Thus, high values at CpG sites indicate hypermethylation over time. The relationships between FLI and methylation levels of CpG sites were determined by calculating the correlation coefficients and *p*-values for all CpG sites. Correlation plots were drawn between FLI and CpG methylation levels in the top nine squares of the correlation coefficients (Figure 4a). Correlation coefficients and *p*-values were calculated for 9256 genes, and 4133 and 5123 genes had positive and negative correlations, respectively, with FLI.

The correlation coefficients of genes were then used to obtain enriched GO (Figure 4b,c) and KEGG terms (Figure 4d). Network analysis was performed, and five nodes were found to be connected to these genes. The closer to red, the higher the beta value, and the FLI and the closer to blue, the lower the FLI (Figure 4e).

### 3.5. Identification of Differently Changed Regions

**Figure 5:**
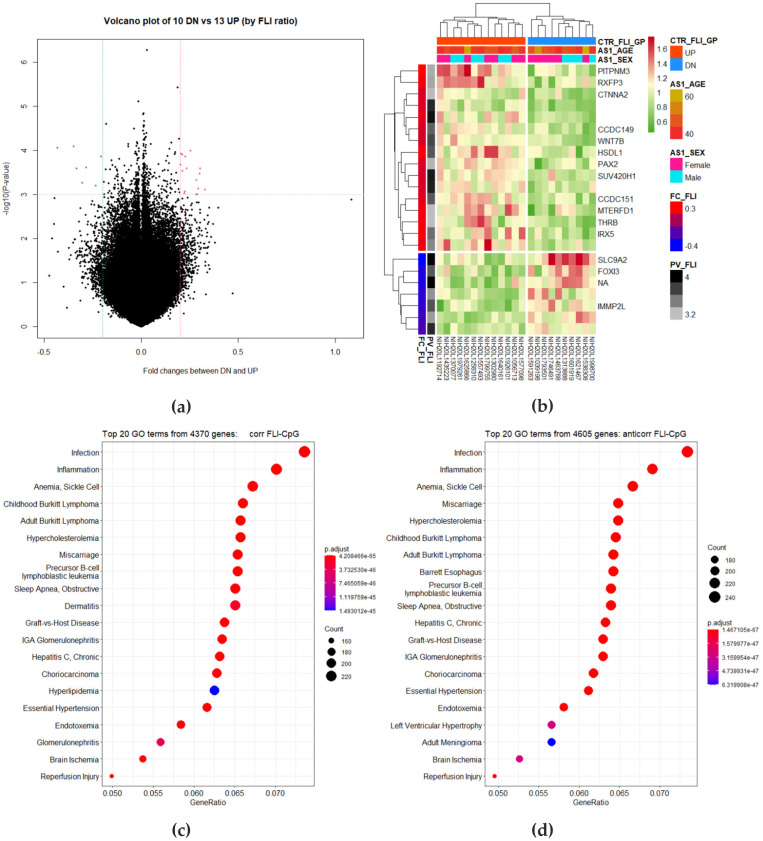

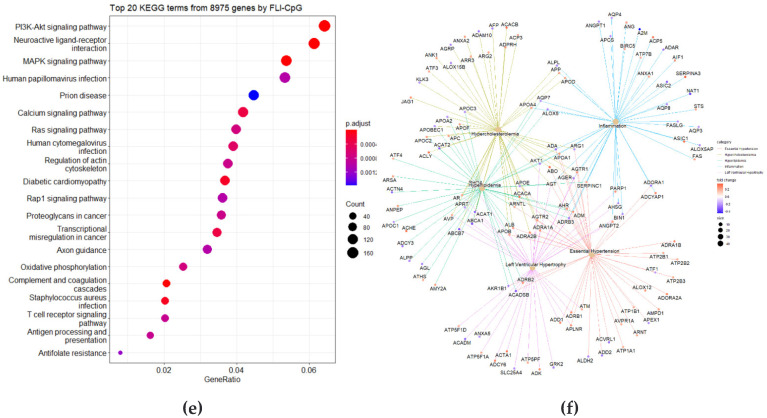
Visualization of differentially methylated regions (DMRs) between the 13 up and 10 down longitudinal changes in fatty liver index (FLI) groups. The methylation level and FLI of the fifth follow-up were divided by that of the first. *p*-values and fold changes (FCs) were retrieved from the *t*-test for the high and low fatty liver index (FLI) groups. (**a**) In the volcano plots, total probes are plotted by FC and *p*-value. The condition is |FC| > 0.2, *p*-value < 0.001. (**b**) A heatmap is presented for 16 increased probes and 7 decreased probes in FLI high groups over time. (**c**–**e**) Enriched gene ontology (GO) terms and Kyoto Gene and Genome Encyclopedia (KEGG) pathways for the high and low FLI groups. The color of the plot represents the *p*-value and the size of the count of genes. (**f**) Gene concept network for correlation coefficients is provided. In each network analysis, the top five terms are listed, and related genes are connected. Color scale of the related genes stands for the FCs. Red and green points indicate increased and decreased genes in the high FLI group over time.

Two groups, 13 up and 10 down longitudinal changes in FLI, were compared by longitudinal changes in CpG sites. A *t*-test was performed between the FLI up and down groups for all the 431,651 CpG sites. If the FC of the analysis result is a positive value, it indicates hypermethylation in the FLI up group over time, and a negative value indicates hypermethylation in the FLI down group. Sixteen CpG sites were selected on the basis of the condition of *p*-value < 0.001 and FC > 0.2, and seven CpG sites were selected on the basis of the condition of FC < −0.2. Selected CpG sites were visualized as volcano plots and heat maps (Figure 5a,b).

A total of 8975 specimens located at the CpG site were selected, and GO analysis was performed on 4370 specimens that had a positive correlation with the amount of change in FLI and 4605 specimens that had a negative correlation (Figure 5c,d). The KEGG enrichment test was also performed and is presented as a dot plot (Figure 5e). As a result of network analysis of genes with a correlation between FLI and methylation change, enrichment terms are presented in a total of five nodes (Figure 5f).

### 3.6. Integration of Four Analyses as Circos Plot

**Figure 6:**
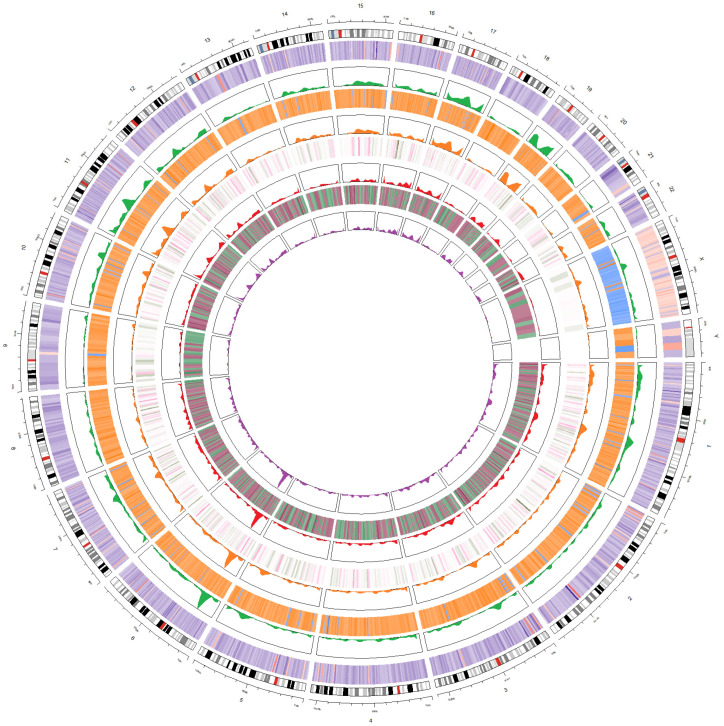
Circos plot. A total of five heatmaps and peaks are listed. High and low fatty liver index were classified by 30. (First from the outside) As a result of *t*-test analysis according to the high and low FLI, fold change and −log10(*p*-value) are presented as heatmaps and peaks. (Second from the outside) As a result of correlation analysis with FLI, r value and −log10(*p*-value) are presented as heatmaps and peaks. (Third from the outside) As a result of *t*-test analysis according to the increased and decreased FLI, fold change and −log10(*p*-value) are presented as heatmaps and peaks. (Fourth from the outside) As a result of correlation analysis with longitudinal changes of FLI, r value and −log10(*p*-value) are presented as heatmaps and peaks.

The four analyses were integrated as heatmaps and peaks in one Circos plot (Figure 6). The Circos plot has eight tracks, heatmaps indicating fold changes or correlation coefficients, and peaks indicating −log10(*p*-value). The four tracks located outside the Circos plot are the results of the correlation and *t*-test analysis for 322 subjects at baseline. The four tracks located inside are the results of the correlation and *t*-test analysis for the 33 people included in the longitudinal study.

In the four analyses, the most statistically significant region was identified in the short arm of chromosome 6. The most significantly differentially expressed genes in each of the four analyses were *FUT9*, *GSTA4*, *L3MBTL3*, and *CD83*. A significant pattern was observed for chromosomes 17 and 19. Meanwhile, in the baseline analysis, a pattern different from the fold-change or correlation coefficient of the autosome was observed on the X chromosome. As a result of the *t*-test analysis at baseline, a high overall fold-change was observed in the X chromosome, and the correlation coefficient with FLI was negative.

## 4. Discussion

The relationship between epigenetic patterns and FLI was revealed in 322 samples simultaneously, and longitudinal epigenetic changes in 33 samples were confirmed. Using two approaches on 322 samples, we retrieved enrichment terms for highly correlated genes and genes located on the DMRs and visualized them as dot plots of KEGG and GO analysis and networks.

Epigenetics, including DNA methylation, plays an important role in the pathogenesis and progression of metabolic disorders, lifestyles, and cancer [34]. However, the mechanisms by which DNA methylation is involved in the development of NAFLD are not fully understood. Previous studies suggest that oxidative stress can change methylation patterns through 8-hydroxydexoyguanosine (8-OHdG) in the liver [35]. Other environmental insults, such as diet-induced hepatic steatosis, can also alter DNA methylation profiles in mouse models [36,37]. The strength of our study is that it elucidates the patterns of DNA methylation associated with FLI under four conditions. Gene and enrichment terms related to different DNA methylation patterns associated with high and low FLI in one-time and longitudinal analyses are presented. A DNA methylation and liver disease model can be developed through a comprehensive analysis of our results and those of previous studies. It has been suggested that overexpression of MMP-9 in cell lines is related to intergenic hypermethylation [38]. Similarly, it is necessary to develop a validation model related to the genes and terms we proposed in future studies.

In the correlation analysis of the change in FLI with time and gene methylation level, performed in the longitudinal follow-up data, the FLI and the methylation level of the *THRB* gene showed a positive correlation. Hepatic thyroid hormone receptor beta, encoded by the *THRB* gene, is involved in systemic lipid regulation and is an important factor in the development and progression of NAFLD. In a gene expression study of human liver biopsy samples, *THRB* expression was negatively correlated with NASH score. This result matches the positive correlation between FLI and *THRB* methylation shown in our study [39]. Hepatic THRB is important because THRB agonists are effective in the treatment of NASH [40]. For example, it was found that the NAFLD activity score improved when Resmetirom (a THR-β agonist) was administered in a mouse model of NASH [41]. Our study demonstrated the significance of thyroid hormone receptors in NASH/NAFLD at the methylation level in a Korean cohort.

We analyzed and selected GO and KEGG enrichment terms from DMRs discovered in 322 subjects, and as a result, various pathways related to FLI were detected. Interestingly, the discovered pathways were hypercholesterolemia and hyperlipidemia. Disruption of cholesterol homeostasis is known to cause hepatocyte damage and steatotic changes and thus contributes to the pathogenesis of NAFLD/NASH [42]. Moreover, thyroid hormones are known to have a strong impact on cholesterol level regulation [43]. Therefore, the relationship between hypercholesterolemia and FLI revealed in this study is consistent not only with the results of previous studies but also with the THRB-related data mentioned above.

Left ventricular hypertrophy was discovered among the enrichment terms from the 33 samples from a longitudinal study. Interestingly, left ventricular hypertrophy associated with NAFLD has been studied in relation to type 2 diabetes mellitus. In particular, *MYO18B* is related to PI3K/AKT/mTOR, a liver cancer-related pathway, and showed a positive correlation with the increase in methylation as the FLI increased over time.

Among the enrichment terms from the two analyses (baseline and longitudinal), hypercholesterolemia and essential hypertension were comorbidities of metabolic syndrome with diabetes, and NAFLD was considered the main risk factor for metabolic syndrome. However, in this study, insulin sensitivity was not selected. Insulin sensitivity plays an important role in the development of NAFLD in metabolic syndrome. This is because the FLI was calculated using limited variables when evaluating NAFLD. In addition, the role of hypercholesterolemia may be overestimated because triglycerides are included in the calculation of the FLI. For the induction and maintenance of insulin resistance, studies related to the mechanism of DNA methylation patterns and related pathways should be conducted.

Factors related to myocardial reperfusion injury have been identified and are related to necroptosis in the steatotic liver. Terms related to anoxia and hypoxia have been discovered and reported to be related to liver disease related to sleep disturbance [44]. As such, we discovered tissue oxygen perfusion- and reperfusion-related factors associated with NAFLD. These findings suggest that NAFLD/NASH is not simply a disease limited to the liver but is also closely related to systemic conditions such as the cardiovascular system.

## 5. Conclusions

In this study, using baseline (*n* = 322) and longitudinal (*n* = 33) cohorts, we extracted FLI-related changes in DNA methylation using *t*-tests and correlation analyses. The association between *THRB* and hypercholesterolemia and essential hypertension has been suggested to be associated with NAFLD.

## 6. Patents

A Korea patent application is pending with similar contents to this study.

## Figures and Tables

**Table 1:** Subject characterization.

Variables	Total Analysis					Baseline				Follow-Up			
	Total (*n* = 322)	Male (*n* = 142)	Female (*n* = 180)	FLI High (*n* = 194)	FLI Low (*n* = 128)	Total (*n* = 33)	Upregulated (*n* = 13)	Similar (*n* = 10)	Downregulated (*n* = 10)	Total (*n* = 33)	Upregulated (*n* = 13)	Similar (*n* = 10)	Downregulated (*n* = 10)
Age	52.56 ± 8.45	51.02 ± 8.17	53.77 ± 8.5	53.39 ± 8.13	51.29 ± 8.8	45.21 ± 5.63	44.69 ± 5.66	43.5 ± 2.32	47.6 ± 7.4	53.15 ± 5.63	52.69 ± 5.57	51.5 ± 2.32	55.4 ± 7.55
Sex	142/180 (44.1/55.9)	142/0 (100/0)	0/180 (0/100)	95/99 (49/51)	47/81 (37/63)	16/17 (48.48/51.52)	6/7 (46/54)	6/4 (60/40)	4/6 (40/60)	16/17 (48.48/51.52)	6/7 (46/54)	6/4 (60/40)	4/6 (40/60)
Diabetes	304/18 (94.41/5.59)	135/7 (95/5)	169/11 (94/6)	180/14 (93/7)	124/4 (97/3)	33/0 (100/0)	13/0 (100/0)	10/0 (100/0)	10/0 (100/0)	29/4 (87.88/12.12)	11/2 (85/15)	8/2 (80/20)	10/0 (100/0)
Hypertension	267/55 (82.92/17.08)	122/20 (86/14)	145/35 (81/19)	144/50 (74/26)	123/5 (96/4)	32/1 (96.97/3.03)	12/1 (92/8)	10/0 (100/0)	10/0 (100/0)	33/0 (100/0)	13/0 (100/0)	10/0 (100/0)	10/0 (100/0)
LipidBlood	318/4 (98.76/1.24)	141/1 (99/1)	177/3 (98/2)	192/2 (99/1)	126/2 (98/2)	33/0 (100/0)	13/0 (100/0)	10/0 (100/0)	10/0 (100/0)	32/1 (96.97/3.03)	12/1 (92/8)	10/0 (100/0)	10/0 (100/0)
BMI	24.52 ± 3.35	24.05 ± 3.28	24.89 ± 3.37	26.16 ± 2.99	22.03 ± 2.12	23.65 ± 2.63	23.26 ± 1.84	24.96 ± 2.87	22.86 ± 2.99	23.56 ± 2.62	23.87 ± 2.18	24.56 ± 2.37	22.17 ± 3.02
Hb	13.56 ± 1.55	14.73 ± 1.14	12.63 ± 1.15	13.92 ± 1.42	13 ± 1.58	13.66 ± 1.42	13.48 ± 1.02	13.76 ± 1.96	13.81 ± 1.37	14.07 ± 1.53	13.94 ± 1.51	14.36 ± 1.81	13.95 ± 1.35
Hematocrit	41.02 ± 4.44	44.48 ± 3.3	38.28 ± 3.12	41.94 ± 4.19	39.62 ± 4.46	41.27 ± 4.24	40.25 ± 3.19	42.1 ± 5	41.75 ± 4.79	41.76 ± 3.83	41.43 ± 3.58	42.49 ± 4.69	41.45 ± 3.49
Platelet	271.96 ± 64.65	260.91 ± 65.51	280.67 ± 62.78	278.19 ± 64.34	262.51 ± 64.22	260.67 ± 54.27	265.08 ± 64.48	259 ± 41.48	256.6 ± 56.21	246.58 ± 67.66	273.62 ± 87.19	239.1 ± 42.47	218.9 ± 48.65
AST	27.93 ± 9.7	30.18 ± 11.16	26.15 ± 7.97	29.45 ± 10.78	25.62 ± 7.24	26.61 ± 8.33	30.31 ± 9.64	24.7 ± 6.63	23.7 ± 6.73	26.88 ± 8.27	28.92 ± 10.9	24.9 ± 8.31	26.2 ± 2.2
ALT	27.07 ± 16.71	32.62 ± 20.38	22.69 ± 11.39	31.47 ± 18.49	20.41 ± 10.58	26.67 ± 12.54	29.38 ± 12.98	26 ± 11.6	23.8 ± 13.4	26.58 ± 14.63	29.54 ± 14.81	28.5 ± 19.58	20.8 ± 5.61
r-GTP	28.41 ± 32.58	41.98 ± 43.1	17.71 ± 13.37	37.66 ± 38.54	14.39 ± 9.86	30.48 ± 27.51	37.31 ± 32.85	33.2 ± 29.68	18.9 ± 12.08	NA	NA	NA	NA
Bilirubin	0.61 ± 0.35	0.72 ± 0.41	0.53 ± 0.26	0.61 ± 0.34	0.62 ± 0.37	0.88 ± 0.41	0.95 ± 0.54	0.84 ± 0.27	0.82 ± 0.37	NA	NA	NA	NA
Creatinine	0.84 ± 0.17	0.96 ± 0.17	0.75 ± 0.11	0.86 ± 0.18	0.81 ± 0.16	0.93 ± 0.2	0.92 ± 0.2	0.94 ± 0.18	0.94 ± 0.24	0.96 ± 0.16	0.97 ± 0.17	0.95 ± 0.12	0.95 ± 0.19
CRP	0.2 ± 0.33	0.19 ± 0.22	0.2 ± 0.39	0.22 ± 0.28	0.17 ± 0.39	0.18 ± 0.22	0.11 ± 0.1	0.31 ± 0.34	0.14 ± 0.07	2.98 ± 7.98	3.91 ± 10.85	4.28 ± 7.69	0.47 ± 0.46
HDL	44.15 ± 9.1	43.35 ± 9.15	44.77 ± 9.04	41.59 ± 7.91	48.02 ± 9.45	46.06 ± 7.66	45 ± 5.74	45.6 ± 9.24	47.9 ± 8.57	46.64 ± 10.39	41.15 ± 6.04	44.6 ± 8.33	55.8 ± 11.15
TG	157.86 ± 91.54	165.3 ± 104.61	151.99 ± 79.54	191.65 ± 100.03	106.64 ± 39.77	129.52 ± 77.49	123.85 ± 50.05	143.2 ± 125.7	123.2 ± 43.74	147.27 ± 131.55	205.15 ± 186.48	130.6 ± 71.83	88.7 ± 32.22
Glu0	91.55 ± 27.85	94.96 ± 26.51	88.86 ± 28.65	97.11 ± 30.72	83.13 ± 20.19	87.58 ± 12.64	86.69 ± 9.65	89.4 ± 19.34	86.9 ± 8.05	99.61 ± 33.58	93.38 ± 7.15	116.6 ± 58.5	90.7 ± 7.33
Drinking	5 ± 15.43	10.84 ± 21.82	0.39 ± 1.95	6.85 ± 19.06	2.2 ± 6.07	6.36 ± 9.98	6.19 ± 10.78	8.35 ± 10.56	4.58 ± 8.94	7.54 ± 14.18	6.53 ± 13.43	11.3 ± 15.84	5.09 ± 14.12

## Data Availability

KoGES dataset information and data shearing processes can be obtained from the site provided by the National Research Institute of Health, Korea Disease Control and Prevention Agency, Ministry for Health and Welfare, Korea (https://nih.go.kr/contents.es?mid=a50401010100, accessed on 25 February 2022). R source code will be provided upon request.
